# ISG15 Is Critical in the Control of Chikungunya Virus Infection Independent of UbE1L Mediated Conjugation

**DOI:** 10.1371/journal.ppat.1002322

**Published:** 2011-10-20

**Authors:** Scott W. Werneke, Clementine Schilte, Anjali Rohatgi, Kristen J. Monte, Alain Michault, Fernando Arenzana-Seisdedos, Dana L. Vanlandingham, Stephen Higgs, Arnaud Fontanet, Matthew L. Albert, Deborah J. Lenschow

**Affiliations:** 1 Department of Medicine, Department of Pathology and Immunology, Washington University School of Medicine, St. Louis, Missouri, United States of America; 2 Department of Immunology, Unité Immunobiologie des Cellules Dendritiques, Institut Pasteur, Paris, France; 3 INSERM U818, Paris, France; 4 Laboratoire de Microbiologie, Groupe Hospitalier Sud Réunion, Saint-Pierre, France; 5 Unité de Pathogénie Virale Moléculaire, Institut Pasteur, Paris, France; 6 CNRS URA 3015, Paris, France; 7 Department of Pathology, University of Texas Medical Branch, Galveston, Texas, United States of America; 8 Biosecurity Research Institute, Kansas State University, Manhattan, Kansas, United States of America; 9 Epidemiology of Emerging Infectious Diseases, Institut Pasteur, Paris, France; Mount Sinai School of Medicine, United States of America

## Abstract

Chikungunya virus (CHIKV) is a re-emerging alphavirus that has caused significant disease in the Indian Ocean region since 2005. During this outbreak, in addition to fever, rash and arthritis, severe cases of CHIKV infection have been observed in infants. Challenging the notion that the innate immune response in infants is immature or defective, we demonstrate that both human infants and neonatal mice generate a robust type I interferon (IFN) response during CHIKV infection that contributes to, but is insufficient for, the complete control of infection. To characterize the mechanism by which type I IFNs control CHIKV infection, we evaluated the role of ISG15 and defined it as a central player in the host response, as neonatal mice lacking ISG15 were profoundly susceptible to CHIKV infection. Surprisingly, UbE1L^−/−^ mice, which lack the ISG15 E1 enzyme and therefore are unable to form ISG15 conjugates, displayed no increase in lethality following CHIKV infection, thus pointing to a non-classical role for ISG15. No differences in viral loads were observed between wild-type (WT) and ISG15^−/−^ mice, however, a dramatic increase in proinflammatory cytokines and chemokines was observed in ISG15^−/−^ mice, suggesting that the innate immune response to CHIKV contributes to their lethality. This study provides new insight into the control of CHIKV infection, and establishes a new model for how ISG15 functions as an immunomodulatory molecule in the blunting of potentially pathologic levels of innate effector molecules during the host response to viral infection.

## Author Summary

Type I interferon plays a critical role in the host defense to viral infection. Signaling through the type I IFN receptor allows for the induction of hundreds of interferon stimulated genes (ISGs) that generate an antiviral state within host cells. The ubiquitin-like molecule ISG15 has been shown to play an important role during multiple viral infections, including influenza virus infection. To date, the ability of ISG15 to protect against viral infection has been shown to be dependent on its ability to covalently bind (or conjugate) to target proteins, including the binding of viral proteins. We investigated the importance of the type I interferon response and ISG15 conjugation in a neonatal model of Chikungunya virus infection, a re-emerging human pathogen in the Indian Ocean region. Remarkably, the role of ISG15 during CHIKV infection appears to be conjugation independent, suggesting a non-classical role for ISG15 during viral infection. Our data also suggests that ISG15 plays an immunoregulatory role, as opposed to having direct antiviral function. Our CHIKV model may provide an opportunity to identify a novel mechanism by which ISG15 contributes to the innate immune response to viral infection.

## Introduction

Chikungunya virus is a member of the genus Alphavirus, which are enveloped positive-strand RNA viruses transmitted by mosquitoes. It was first isolated in Tanzania in 1952, and reported to cause severe fever as well as myalgia, joint pain and rash within 2–5 days of infection [1], [2]. Recently, CHIKV reemerged in Eastern Africa and has developed into a major epidemic in the Indian Ocean region. In 2006, an outbreak on the island of La Réunion resulted in the infection of approximately one-third of the inhabitants [3], [4]. It has since spread to India and Southeast Asia with estimates of between 1–6 million people having been infected [5], [6]. Concerns for the globalization of this virus has evolved given the continuation of this epidemic, the high serum viremia seen in infected patients, and mutations in the currently circulating strain of CHIKV that have allowed it to adapt to a more widely distributed mosquito vector [7]. These concerns have been raised by both an increase in the number of foreign travelers contracting CHIKV and returning to both Europe and the United States, and by the possibility of spread by infected individuals, the latter being exemplified by an outbreak in Italy in 2007 and in Southern France in 2010 [5], [8], [9].

The typical clinical presentation of adults infected with CHIKV includes fever, rash, arthralgias and severe myalgias. Infected neonates, however, display more severe disease, with symptoms including encephalopathy and cerebral hemorrhage, with a subset of these infants developing permanent disabilities [10]. During the recent epidemic, it was reported for the first time that CHIKV-infected mothers can transmit the virus to their newborns during delivery, with a vertical transmission rate of approximately 50%, and in some instances infection resulted in mortality [10]. This age dependence of disease severity has also been reported for other alphaviruses and can be reproduced in mouse models through the infection of suckling mice. Indeed, it has been shown that neonatal mice succumb to Ross River Virus (RRV) [11], Semliki Forest Virus (SFV) [12], Sindbis Virus (SINV) as well as CHIKV [13]–[15]. There are likely several factors that contribute to this increased sensitivity in neonates, including alterations in the neonatal immune response.

Regarding immune function in neonates, developmental delays have been described for the adaptive immune responses, however less is known about neonatal innate responses. While still controversial, many reports indicate that neonatal responses are diminished as compared to adults. For example, cord blood cells stimulated with toll-like receptor (TLR) ligands produced low levels of TNFα, IL-1β, and IL-12 [16]. Neonatal plasmacytoid dendritic cells (DCs) have also been shown to have impaired production of type I IFN in response to CpG stimulation [17]. It has been shown that LPS does not effectively activate IRF-3 dependent responses, including the production of IFNβ [18]. The response to cytokines may also be impaired as there is evidence that STAT-1 recruitment following IFNγ stimulation is less efficient in neonatal than in adult leukocytes [19]. Based on the critical role of type I IFN in the control of CHIKV infection [20], we considered the possibility that neonates may have a developmental delay in their type I IFN response, possibly contributing to their increased susceptibility to CHIKV infection and viral dissemination.

In response to CHIKV infection, the production of type I IFNs is triggered by the engagement of the Rig-I like receptor (RLR) pathway [20], [21]. IFNs then stimulate the induction of hundreds of interferon stimulated genes (ISGs) and it is through these ISGs that IFNs mediate their effector function [22]. One of the earliest ISGs induced following IFN stimulation is ISG15. ISG15 is a 17 kDa protein that contains two ubiquitin-like domains connected by a proline peptide linker [23]. Similar to ubiquitin, ISG15 can form conjugates with an array of intracellular host and viral proteins through the use of an E1 (UBE1L), E2 (UbcH8), and E3 (e.g., Herc5, EFP, HHARI) enzymatic cascade [24]–[28]. Conjugation of ISG15 to target proteins has been suggested to cause either a gain or loss of function of the targeted protein, although the consequences of ISG15 conjugate formation are not well understood [29]–[32]. An unconjugated form of ISG15 can also be found in the sera of humans treated with IFN-β_ser_ as well as in virally infected mice [33], [34]. The released form of ISG15 has been suggested to have cytokine like activity [35]–[37], however its role during an antiviral immune response has not been examined.

The rapid induction of ISG15 following IFN stimulation has led to the identification of an antiviral role for ISG15 during infection. ISG15^−/−^ mice display increased susceptibility to SINV, herpes simplex virus-1 (HSV-1), gamma herpes virus (γHV68), influenza A and B viruses, and vaccinia virus [34], [38]. ISG15^−/−^ mice infected with influenza B virus display a 2–3 log increase in lung viral titers as well as elevated cytokine and chemokine levels [34]. The ability of ISG15 to conjugate to target proteins appears to be essential for ISG15's antiviral activity during certain viral infections, as UbE1L^−/−^ mice, which lack the ability to form ISG15 conjugates, phenocopy ISG15^−/−^ mice during both SINV and influenza B virus infection [39]–[41]. Further support for the importance of ISG15 conjugation during viral infection comes from the evolution of viral proteins that directly target ISG15 conjugate formation. Both the NS1 protein of influenza B virus and the E3L protein of vaccinia virus inhibit ISG15 conjugate formation, while OTU-domain containing viral proteins, such as the L protein of Crimean-Congo hemorrhagic fever virus (CCHFV) or the Nsp2 protein of Equine Arteritis virus (EAV), and the SARS coronavirus papain-like protease (SARS-CoV PLpro) exert both deubiquitinating and deISGylating activity [24], [38], [42], [43]. Therefore, the antiviral activity of ISG15 has been thought to be conjugation dependent.

Herein we demonstrate that despite an increased susceptibility of neonates to CHIKV infection, they produce robust levels of type I IFNs. While insufficient to completely control infection, IFNs participate in limiting the infection. We also show that ISG15 is induced during CHIKV infection and plays a critical role in protecting neonatal mice from viral induced lethality. Surprisingly, the mechanism of action by which ISG15 limits infection is independent of UbE1L mediated conjugation, as UbE1L^−/−^ mice displayed no phenotypic differences as compared to WT animals. Furthermore, ISG15 does not directly inhibit viral replication, as suggested by the similar viral loads in WT and ISG15^−/−^ mice. Instead, ISG15 appears to function as an immunomodulatory molecule in this model. These data demonstrate a novel role for ISG15 during viral infection and suggests that prophylactic measures targeting the induction of IFN and ISG15 may help protect neonates during future CHIKV outbreaks.

## Results

### CHIKV infected infants produce high levels of IFN and IFN-induced chemokines/cytokines

Based on the increased severity of neonatal disease that has been observed during the recent epidemic of CHIKV, we assessed the inflammatory response of infants during the acute phase of CHIKV infection. Patients were recruited at the time of presentation in the emergency room and sera samples were collected and stored. CHIKV infection was confirmed by RT-PCR; and all patients were negative for anti-CHIKV IgG and IgM, indicating acute infection. We performed multi-analyte testing using Luminex technology, with a focus on inflammatory cytokines and chemokines. The inflammatory signature was compared to uninfected patients presenting to the emergency room for reasons unrelated to acute infection (e.g., broken bone). The intensity of the immune response in the infant vs. adult cohorts was compared. Non-parametric tests were used for the statistical analysis, and a false discovery rate (FDR) correction was applied to all p-values in order to adjust for multiple testing.

**Figure 1:**
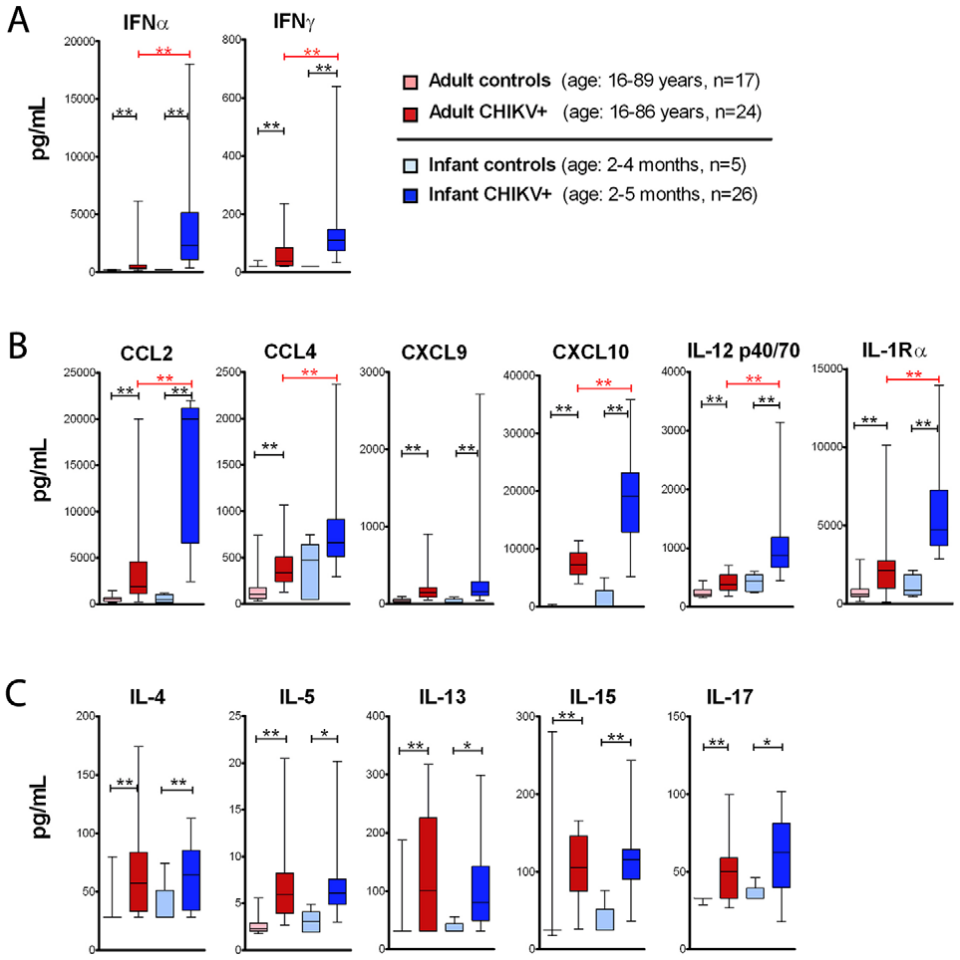
Serum concentration of IFN and IFN-induced chemokines is higher in CHIKV infected infants as compared to adults. Patient sera were obtained from acute CHIKV infected adults (age = 16–86 years) and infants (age = 2–5 months). Age matched controls from La Réunion were tested. Chemokines / cytokines were measured using a Luminex assay. (**A**) IFNα and IFNγ levels; (**B**) IFN-induced molecules; and (**C**) Lymphocyte cell derived cytokines are shown. Whisker-box plots are shown (line indicates median; boxes represent first and third quartile; and bars define range). Mann-Whitney U-test was performed using a false discovery rate (FDR) procedure for generating corrected p-values. Comparisons were made between CHIKV patients versus their control cohort (in black); as well as adult versus infant patients (in red). ** indicates p<0.005, * indicates p<0.05. IFNα data from adult patients were previously reported [20], but are shown here for comparison to data from infected neonates.

We detected elevated levels of both IFNα and IFNγ in both groups of patients when compared to their respective control group (p<0.005). Interestingly, both IFNα and IFNγ levels were more elevated in infants in comparison to adult patients (**Figure 1A**). In addition, the chemokines/cytokines known to be induced by IFNs were highly expressed. These included CCL2, CCL4, CXCL9, CXCL10, IL-1Rα, IL-12p40/p70; and strikingly these analytes, with the exception of CXCL9, showed higher plasma concentrations in infants as compared to adult patients (**Figure 1B**). Despite the clinical presentation including fever (**Table S1**), levels of the pyrogenic cytokines IL-1β and TNFα were not significantly elevated in patients when compared to their controls; with only IL-6 being slightly upregulated in adult patient as compared to the control group (data not shown). Also of interest, while we observed marked induction of so-called Th1 cytokines, such as IFNγ and IL-12p40/p70, there was no clear skewing toward a Th1 response. In fact, we observed high levels of Th2 cytokines as well as Th17 cytokines (**Figure 1C**). Notably, these interleukins were upregulated as compared to healthy individuals, but were similarly expressed in the infected infant and adult groups. These data suggest that infants, while developing more severe manifestations of CHIKV, do indeed mount a robust acute response to infection.

**Figure 2 ppat-1002322-g002:**
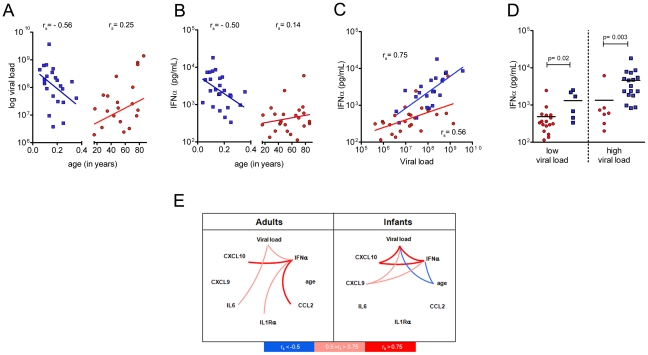
Higher serum IFNα in infants is not explained by differences in viral load. (**A–C**) Correlations between viral load vs. age; IFNα vs. age; and IFNα vs. viral load were evaluated. CHIKV infected infants are represented by blue squares; CHIKV infected adults are represented by red circles. Lines indicate linear regression and Spearman correlation (r_s_) values are shown. (**D**) Each group was divided into two subgroups (<median: low viral load or >median: high viral load; Median = 4.8×10^7^) and statistic are represented (Mann Whitney test) (**E**) For analytes identified in Figure 1 that showed a correlation with viral load, IFN or age are plotted in a network array, illustrating the correlations identified in neonatal *vs.* adult individuals. Connecting lines indicate Spearman correlation (r_s_) values; positive correlations in red and negative correlations are depicted in blue. IFNα data from adult patients were previously reported [20], but are shown here for comparison to data from infected neonates.

One potential caveat to this conclusion is that the viral load is higher in infants, possibly accounting for greater immune activation (median viral load in infants = 1.6×10^8^ RNA copies; median viral load in adults = 1.4×10^7^ RNA copies). Within the infant group, viral load negatively correlated with age, corroborating the observation that newborns are more susceptible to CHIKV induced disease (**Figure 2A**). In adults, there was also an age-dependent trend with elderly harboring higher viral loads (**Figure 2A**). Again this is consistent with the report that increased age is a risk factor for severe CHIKV disease [44]. Next, we plotted plasma IFNα concentrations as a function of age. These data demonstrate a strong negative correlation in infants, and followed the pattern seen for CHIKV titers (**Figure 2B**). We found that IFNα levels correlated with viral loads in both group (**Figure 2C**). To normalize for CHIKV titers, we separated each cohort in two groups (<global median: low viral load; and > global median: high viral load); in both groups the levels of IFNα were more elevated in infected infants than in infected adults (**Figure 2D**). Furthermore, we performed univariate linear regression analysis to model the effect of infant status on plasma IFNα concentrations. Infants had higher IFNα concentrations compared to adults (RGM [95% CI] = 5.49 [3.16–9.53]; p<0.001). This effect remained significant after adjustment for viral load (adjusted RGM [95% CI] = 3.44 (2.07–5.70]; p<0.001). Thus for a similar viral load, infants produce more IFNα than adults. To represent associations between age, viral load, IFNα and the immune signature, we established a network plot of significant correlations (**Figure 2E**). These data argue against infants being compromised in their response to CHIKV infection; however, we could not determine whether their IFN response was protective. To address this issue and to examine the mechanism of ISG mediated control of CHIKV, we exploited a recently described neonatal mouse model for studying infection [15].

### CHIKV infection in neonatal mice results in a robust type I interferon and proinflammatory cytokine response that is critical in controlling infection

**Figure 3:**
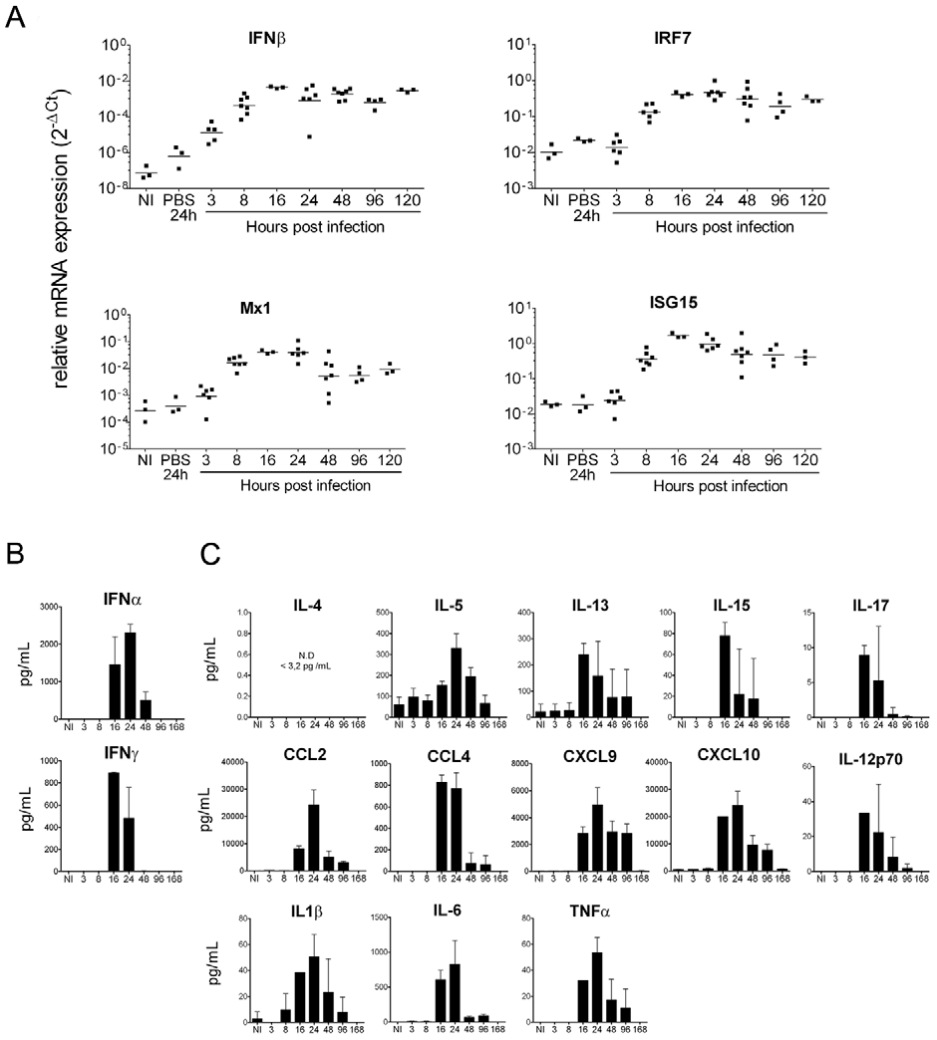
Neonatal mice mount a robust IFN and pro-inflammatory cytokine/chemokine response following CHIKV infection. Nine day old C57BL/6 mice were injected s.c. in the right flank with CHIKV. (**A**) The skin at the site of infection was harvested at the indicated times post infection and IFNβ, IRF7, Mx1 and ISG15 gene expression was evaluated by qRT-PCR. The ΔCt was calculated using GAPD as a reference gene. At various time points, sera was harvested and tested for (**B**) IFNα by ELISA and IFNγusing Luminex. (**C**) Serum cytokines and chemokines were also analyzed using Luminex. Each graph represents 2–3 independent experiments with 3 mice per experiment.

Previously, we reported an age-dependent susceptibility to CHIKV infection in mice. Infection of 6 day old animals resulted in 100% mortality; 9 day old animals developed paralysis, with approximately 50% of the animals succumbing to infection; while by 12 days of age the mice became refractory to symptoms of severe disease and lethality [15]. To compare our experimental mouse model of CHIKV infection to the response seen in human infants we assessed the IFN and proinflammatory responses in 8–9 day old mice. We first assessed the induction of the IFN response at the local site of infection by monitoring mRNA levels of IFNβ and selected interferon stimulated genes (ISGs) in the skin. Mice were inoculated with 2×10^5^ PFU of CHIKV and the injection site was removed between 3–120 hrs post-infection. Increased expression of IFNβ mRNA could be detected as early as 3 hrs post-infection with peak levels being achieved at 16 hrs post-infection (**Figure 3A**). Similar to IFNβ mRNA induction, IRF7, Mx1 and ISG15 mRNA levels could also be detected as early as 3 hrs post-infection, with peak levels observed at 16 hrs post-infection (**Figure 3A**). Thus, at the site of infection, neonatal mice are able to induce IFNβ expression as well as a subset of known ISGs. Of note, IRF7 and Mx1 mRNA expression is indicative of signaling via the type I IFN receptor, suggesting that the production as well as reception of IFNαβ is intact in neonatal mice.

We next assessed the systemic inflammatory response in this model. Similar to our findings in human infants infected with CHIKV, we observed a strong induction of IFNα and IFNγ in infected pups (**Figure 3B**). Plasma concentration of CCL2, CCL4, CXCL9 and CXCL10 were also elevated, however IL-12p70 was only modestly induced (**Figure 3C**). Similar to the human data, a mixed Th1, Th2 and Th17 cytokine profile was observed with the induction of IL-12, IL-5, IL-13, IL-15, and IL-17 (**Figure 3C**). For all analytes, peak levels were seen 16–24 hrs post-infection (**Figure 3**). Notably, there were some differences seen between the murine neonate and human infant inflammatory profiles. Most interestingly, the mice displayed increased levels of the pyrogenic cytokines, including IL-1β, IL-6 and TNFα, which were not seen in our studies of human infants (**Figure 3C**). While this may represent differences in pathogenesis, we believe it is more a reflection of the fact that the mice can be assessed within hours of viral inoculation, while the exact timing of the human infection is unknown. Overall, we find that the similarities between the mouse and human responses support the use of neonatal mice to study the response to CHIKV infection, and indicate that induction of IFNs, as well as the triggering of an ISG response, are both rapid and robust.

**Figure 4:**
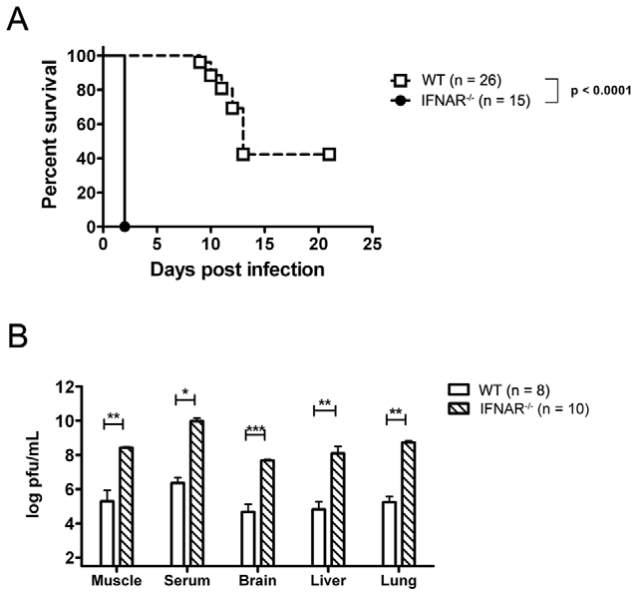
Endogenous Type I IFN is required for the control of neonatal CHIKV infection. WT and IFNAR^−/−^ mice were infected at 9 days of age with 2×10^5^ PFU CHIKV s.c. (**A**) Mice were monitored for lethality for 21 days with data displayed as Kaplan-Meier curves. (**B**) Tissue and sera were collected on day 1 post-infection and viral titers were determined using a standard plaque assay. Mann-Whitney statistical comparison of WT and IFNAR^−/−^ viral titers are shown where * indicates p<0.05, ** p<0.005 and *** p<0.0005, and vertical bars represent standard error of the mean.

Next, to confirm that endogenous IFN contributes to the control of CHIKV in neonatal mice, mice lacking subunit 1 of the type I IFN receptor (IFNAR^−/−^) mice were infected with CHIKV at 9 days of age. Consistent with our previous observations in adult mice [15], neonatal pups lacking IFNAR1 were highly susceptible to CHIKV infection with 100% of the pups dying by day 2 post-infection (**Figure 4A**). These pups developed a rapid, disseminated infection. Within 1 day of infection, the IFNAR^−/−^ mice displayed viral loads at the injection site that were 100-fold higher than that detected in WT controls. We also observed a striking increase in viral titers in the serum and multiple organs, including the brain, liver, and lung (**Figure 4B**). These data indicate that endogenous IFN, while insufficient to protect mice, plays an important role in limiting CHIKV infection during disease pathogenesis in neonatal animals.

### Pre-treatment with Poly I∶C prevents clinical disease and CHIKV induced death

**Figure 5:**
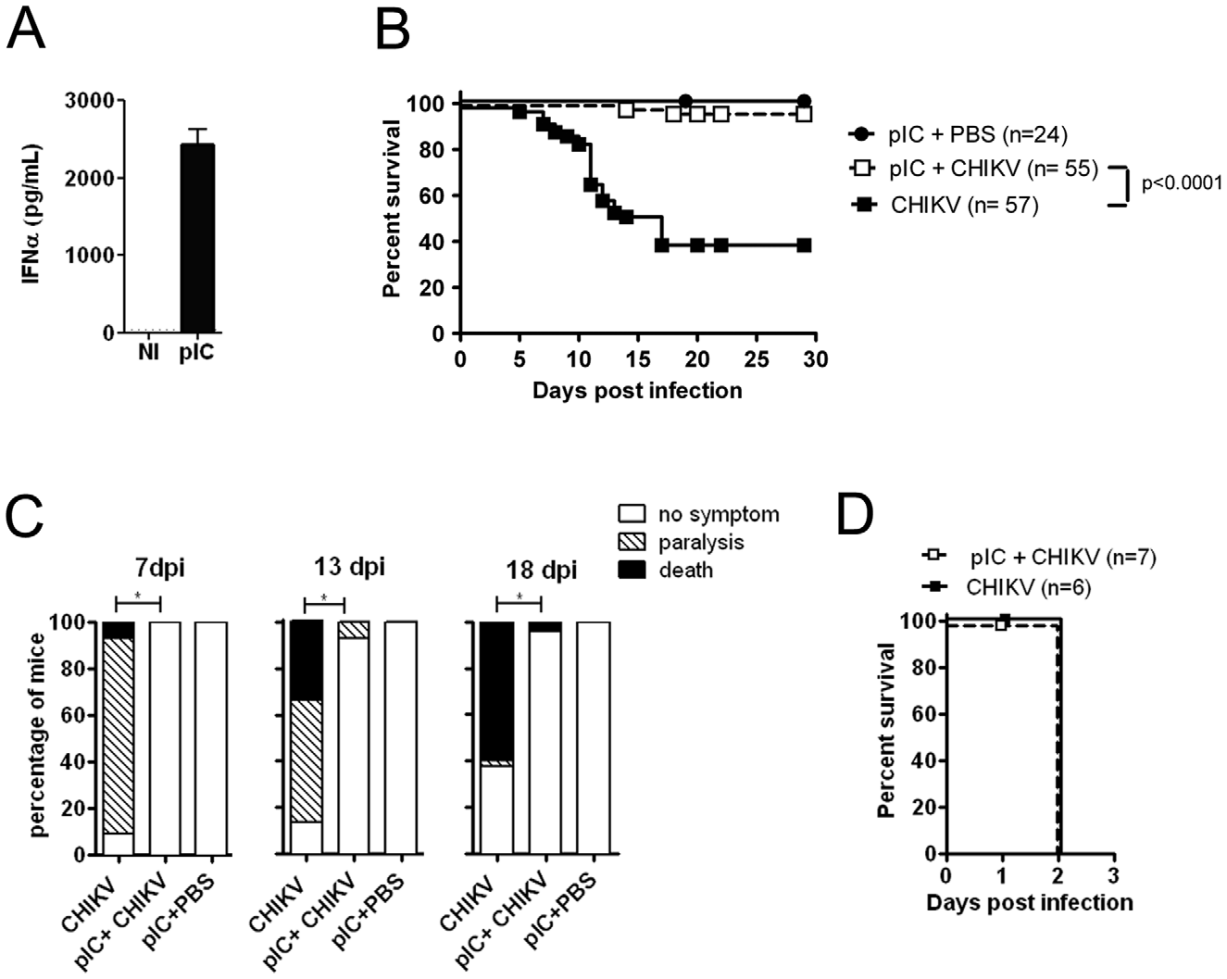
Adjuvant induced type I IFN production protects neonates from CHIKV infection. Mice (8 days of age) were injected i.p. with 25 µg of pIC and 24 hours later mice were infected s.c. in the right flank with CHIKV. (**A**) IFNα levels produced in the serum of neonatal mice 7 hours post injection with 25 µg pIC. (**B**) Survival was monitored daily for four weeks and displayed as Kaplan-Meier survival curves. (**C**) Mice were scored for clinical signs of disease on days 7, 13 and 18 post-infection as discussed in materials and methods. (**D**) IFNAR^−/−^ mice (8 days of age) were injected i.p. with 25 µg of pIC and 24 hours later mice were infected with CHIKV s.c. Survival was monitored daily.

Our results suggest that signaling via the IFNαβ receptor in neonatal mice results in the robust induction of at least a subset of ISGs and interferon-induced serum proteins (**Figure 3**), and that the absence of IFNAR1 results in exacerbated infection and rapid death (**Figure 4**). One potential explanation is that neonates possess a developmental delay in their IFN-response that makes them susceptible to infection as compared to adult animals. It was therefore important to determine whether prophylactic innate immune responses could protect neonatal animals from CHIKV infection. To evaluate this question, we injected mice with 25 µg of poly I∶C (pIC), a known inducer of type I IFN, and 1 day later mice were challenged with 2×10^5^ PFU of CHIKV. At 7 hrs post treatment with pIC, robust levels of IFNα were detected in the serum of neonatal mice (**Figure 5A**). Following CHIKV infection, mice were monitored daily for the development of symptoms and followed for lethality. Remarkably, 96% of the pIC treated mice survived, in contrast to only 40% of control mice (**Figure 5B**). Treatment with pIC also prevented CHIKV induced paralysis, with only 7% of the pIC treated mice developing paralysis during the course of the infection, as compared to 84% in the control group (**Figure 5C**). Independent experiments were performed using prophylactic treatment with 5000 U IFNβ. At both day 7 and day 11 post-infection, mice receiving recombinant IFNβ displayed less severe disease, as compared to control PBS injected animals (**Figure S1**), however, we did not see a statistical difference in survival, possibly due to the short half-life of IFNβ. To confirm that the protection induced by pIC was mediated by IFN, we repeated these experiments in IFNAR^−/−^ mice. As seen in **Figure 5D**, treatment of IFNAR^−/−^ mice with pIC resulted in no protection from lethality. These results indicate that the prophylactic engagement of the IFN receptor in neonates is able to induce a protective anti-viral response.

### ISG15 plays a significant role in the control of neonatal CHIKV infection

**Figure 6:**
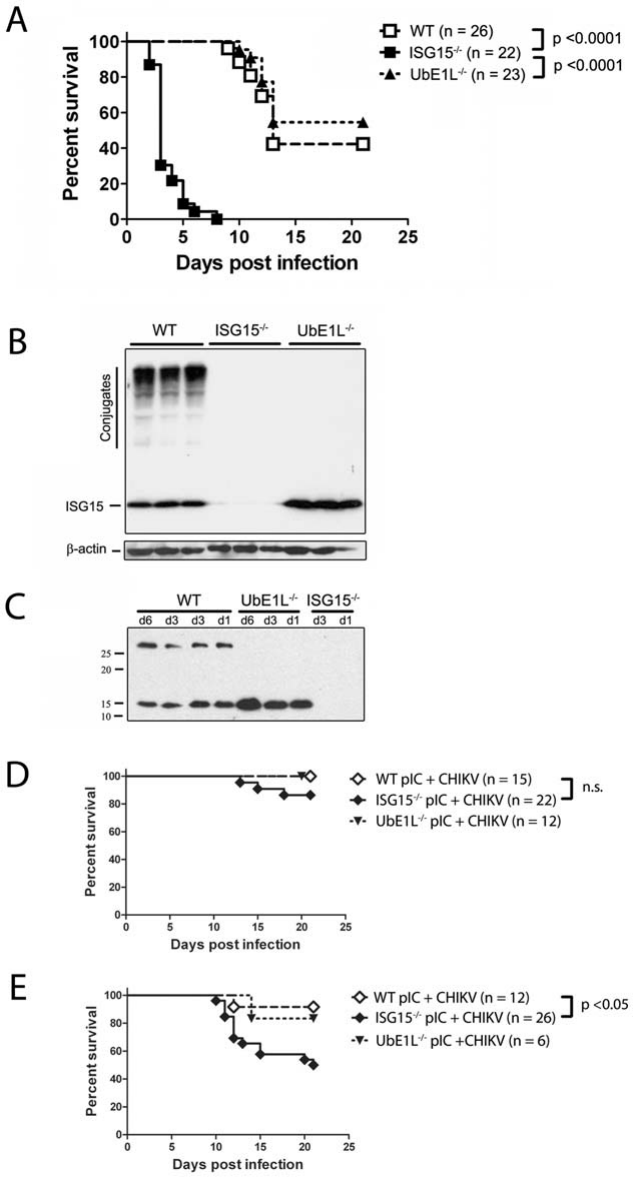
ISG15, independent of UbE1L, plays a critical role during the neonatal response to CHIKV. (**A–C**) WT, UbE1L^−/−^ and ISG15^−/−^ mice were infected with 2×10^5^ PFU CHIKV s.c. at nine days of age. (A) Mice were monitored for lethality for 21 days with data displayed as Kaplan-Meier curves. (B) Skin and muscle homogenates from the site of infection were collected on day 2 post infection and analyzed for ISG15 expression by western blot. (C) Sera collected on days 1, 3 and 6 post-infection and analyzed as in (B). (**D and E**) ISG15^−/−^, UbE1L^−/−^ and WT mice (8 days of age) were injected i.p. with (D) 25 µg or (E) 10 µg of pIC and 24 hours later mice were infected s.c. with 2×10^5^ PFU CHIKV. Survival was monitored daily for 21 days and displayed as Kaplan-Meier survival curves. Mice from all three genotypes that were pretreated with 25 µg of pIC and mock infected with PBS showed no lethality (data not shown).

Interferons mediate their antiviral activity through the induction of ISGs, thus suggesting that investigation of the downstream effector molecules may offer additional insight into how neonates respond to CHIKV infection. The analysis of selected ISGs expressed at the site of infection revealed that ISG15 mRNA was rapidly and strongly induced during CHIKV infection (**Figure 3A**). ISG15 is a ubiquitin-like molecule that conjugates to both host and viral proteins and has been previously shown to participate in the host response to SINV infection. An evaluation of ISG15 protein expression at the site of infection and within the serum confirmed that ISG15 and ISG15 conjugates were induced during CHIKV infection in WT mice (**Figure 6B,C** **).** As we previously described during neonatal SINV infection [45], the expression of ISG15 during CHIKV infection was also dependent upon intact IFN signaling since IFNAR^−/−^ pups infected with CHIKV did not induce detectable levels of ISG15 (**Figure S2**). To test the hypothesis that ISG15 is important in the control of CHIKV, we infected ISG15^−/−^ neonatal mice, comparing them to weight and age-matched WT control animals. Mice were followed daily for signs of illness and survival. As reported above, infection of 9 day old WT neonatal mice resulted in 58% lethality, with deaths occurring between days 10–13 post-infection. In contrast, a dramatic increase in lethality was observed in neonatal mice lacking ISG15, with greater than 70% of the ISG15^−/−^ mice succumbing to infection within 3 days, and 100% of the mice dying by day 9 post-infection (**Figure 6A**).

It has been previously shown that WT mice become resistant to CHIKV induced lethality between 9 to 12 days of age, while mice lacking IFNAR1 remain susceptible to infection even as adults. Since ISG15 is an IFN-induced protein we next determined if its activity was age dependent. We infected either WT or ISG15^−/−^ mice at 11 or 12 days of age, or we infected adult mice between 6–8 weeks of age. By 11 days of age the WT mice had become largely resistant to CHIKV induced lethality with only 15% of the mice succumbing to infection (**Figure S3A**). In contrast, we observed 100% lethality in the ISG15^−/−^ mice, although the onset of lethality was delayed, with the majority of the mice dying between 10 and 14 days post-infection. By 12 days of age the ISG15^−/−^ mice still showed clinical signs of disease, with a dramatic decrease in weight gain as compared to the WT controls (data not shown), but by this age only 20% of the ISG15^−/−^ mice succumbed to the infection (**Figure S3B**). Strikingly, adult ISG15^−/−^ mice, similar to WT controls, displayed no lethality and showed no signs of disease following CHIKV infection (**Figure S3C**). Therefore, we conclude that ISG15 contributes to the control of CHIKV during neonatal infection, but redundant mechanisms are responsible for the control of CHIKV during adult infection.

Since IFNs induce the expression of many ISGs, we next wanted to investigate if ISG15 contributed to the protective anti-viral response established by prophylactic induction of type I IFN. WT or ISG15^−/−^ mice were treated with 10–25 µg of pIC and 1 day later challenged with 2×10^5^ PFU of CHIKV. Not unexpectedly, pretreatment with pIC offered protection to both WT and ISG15^−/−^ mice. After pretreatment with 25 µg of pIC, 14% of the ISG15^−/−^ mice still succumbed to infection as compared to complete protection seen in the WT mice (**Figure 6D**). Interestingly, only 50% of the ISG15^−/−^ mice were protected after pretreatment with 10 µg pIC, as compared to 85% protection observed in the WT mice (**Figure 6E**). These data support our observation that ISG15 is induced as part of the IFN response and that it plays an important role during CHIKV infection.

### ISG15 controls neonatal CHIKV infection independent of UbE1L mediated conjugation

We next investigated the mechanism by which ISG15 protects neonatal mice from CHIKV infection. We had previously shown that the conjugation of ISG15 to target proteins is essential for the control of several viral infections [40], [41]. UbE1L is the only identified E1 for ISG15, and mice lacking UbE1L express free ISG15, but fail to form ISG15 conjugates [39]. To establish a role for ISG15 conjugation, 9 day old UbE1L^−/−^ mice were infected with CHIKV. Surprisingly, UbE1L^−/−^ mice displayed no increase in lethality following CHIKV infection, and instead had a lethality curve similar to that observed in WT mice (**Figure 6A**). Similarly, pIC treated UbE1L^−/−^ mice were protected to the level of WT controls (**Figure 6D, E**). Western blot analysis on skin/muscle from the site of infection (**Figure 6B**), as well as serum (**Figure 6C**), was used to confirm that UbE1L^−/−^ mice generated no ISG15 conjugates during CHIKV infection, whereas WT mice showed robust conjugate formation. Additional organs (lung and liver) were also examined and no conjugates were detected in UbE1L^−/−^ mice (data not shown). Thus, we demonstrate that the activity of ISG15 during CHIKV infection is UbE1L independent.

In an attempt to provide additional evidence that unconjugated ISG15 was functioning in this model, we generated recombinant double subgenomic CHIK viruses that expressed either WT ISG15 (CHIK- LRLRGG) or a mutant, non-conjugatable form of ISG15 (CHIK- LRLRAA) (**Figure S4A**). This strategy was based on our previous report in which recombinant SINV expressing WT ISG15 (LRLRGG) rescued the increased lethality observed in the ISG15^−/−^ mice, while mutant ISG15 (LRLRAA) failed to compensate for the ISG15 deficiency [34], [40]. Both CHIK-LRLRGG and CHIK-LRLRAA expressed ISG15 and displayed similar growth kinetics to CHIK-GFP in BHK cells (**Figure S4B, 4C**). When we infected ISG15^−/−^ mice, however, neither CHIK-LRLRGG nor CHIK-LRLRAA was able to protect ISG15^−/−^ mice (**Figure S4D** and data not shown). While these results do not allow us to confirm that the activity of ISG15 is independent of conjugation, they do suggest that the mechanism of action of ISG15 during SINV and CHIKV infection are distinct.

**Figure 7:**
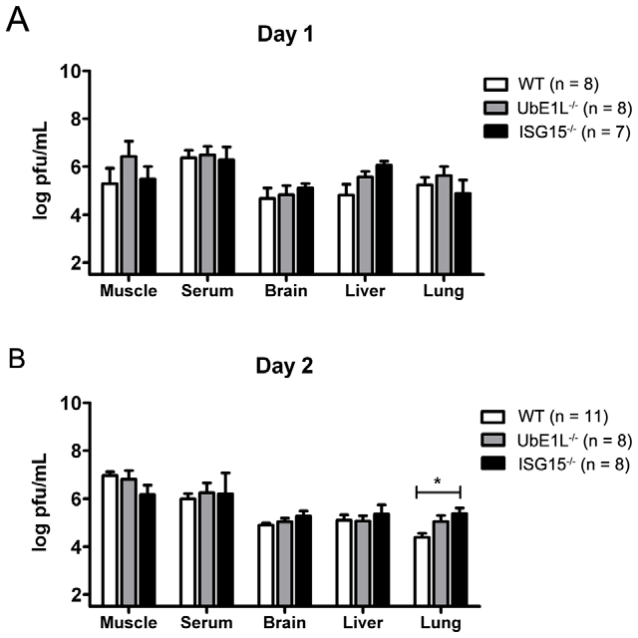
ISG15^−/−^ and UbE1L^−/−^ mice display similar viral loads to WT mice. Nine day old WT, UbE1L^−/−^ and ISG15^−/−^ mice were injected with 2×10^5^ PFU CHIKV s.c. Tissue and sera were collected on days 1 (**A**) and 2 (**B**) post-infection and viral titers were determined by plaque assay; vertical bars represent standard error of the mean. A three way comparison of the three genotypes performed using a Kruskal-Wallis analysis was not significant except for the lung on day 2 post infection (*, where p<0.05).

To further evaluate the mechanism by which ISG15 functions during neonatal CHIKV infection we examined lymphocyte subsets and viral titers in WT, UbE1L^−/−^ and ISG15^−/−^ mice. We detected no significant differences in the lymphocyte subsets of naïve neonatal WT, UbE1L^−/−^ and ISG15^−/−^ mice (**Table S2**). Similar results have been observed in both naïve and pIC stimulated adult mice [39], [45]. These data suggest that differences in starting cell populations do not account for the increase in lethality observed in ISG15^−/−^ mice. We next assessed viral titers on days 1 and 2 post-infection. As expected, UbE1L^−/−^ mice displayed similar viral loads in multiple tissues when compared to WT mice at days 1 and 2 post-infection (**Figure 7A and B**). Given that the ISG15^−/−^ pups infected at 9 days of age displayed a dramatic increase in lethality, with kinetics similar to that observed in the IFNAR^−/−^ mice, we expected to detect increased viral titers in the ISG15^−/−^ mice. To our surprise, at 1 day post-infection the serum and tissues analyzed from the ISG15^−/−^ mice contained viral titers that were similar to those obtained in both UbE1L^−/−^ and WT mice (**Figure 7A**). This was in striking contrast to the 2–3 log increase in viral loads detected in the IFNAR^−/−^ mice 1 day post-infection **(** **Figure 4B** **)**. By 2 days post-infection, just prior to when the majority of ISG15^−/−^ mice succumb to infection, we still detected similar viral loads between WT, UbE1L^−/−^ and ISG15^−/−^ mice in the analyzed tissues (**Figure 7B**). Based on these data we suggest that ISG15 may not be playing a direct anti-viral role, but instead may be acting via an unexpected mechanism of regulating host sensitivity to the viral induced immune response.

**Figure 8:**
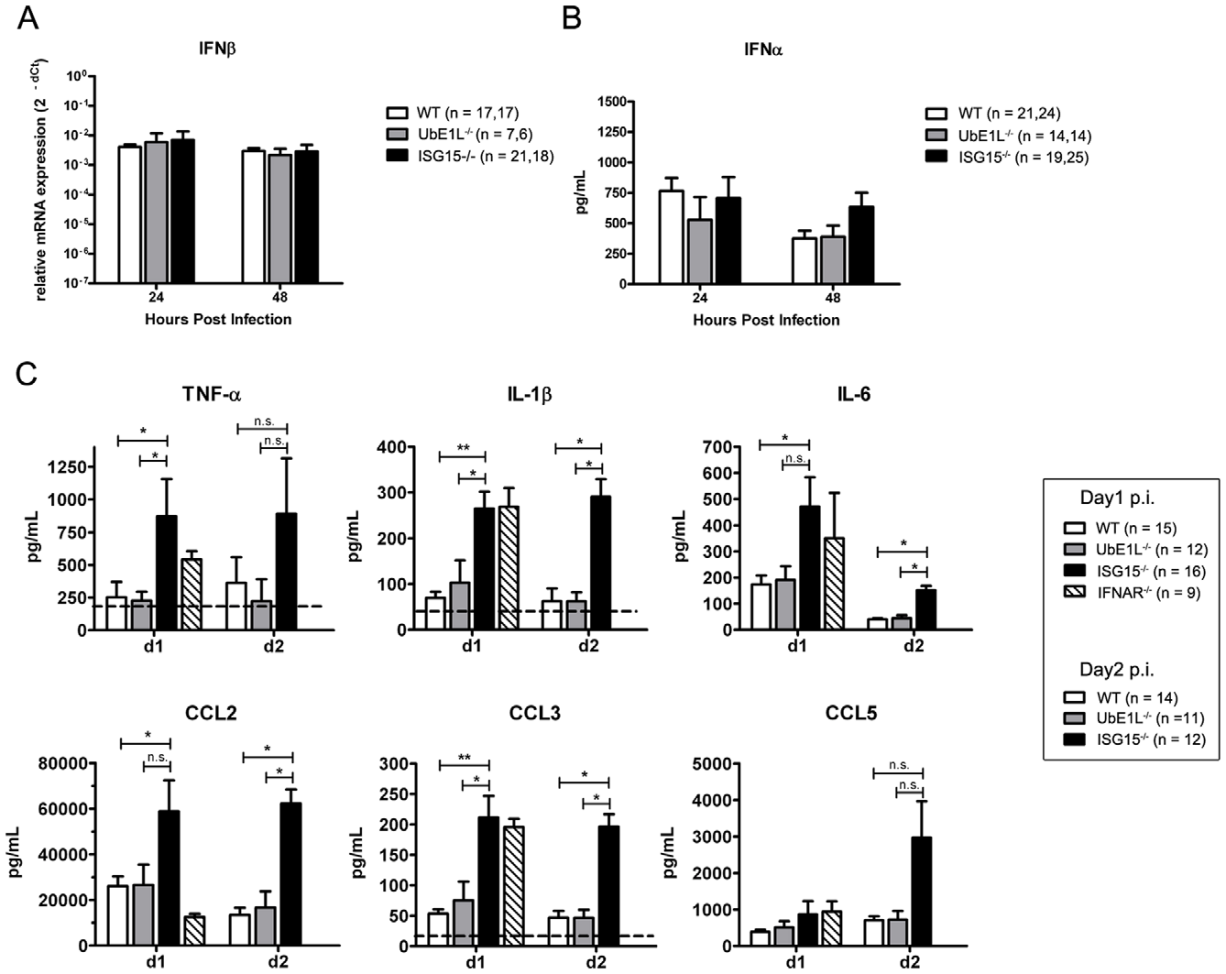
ISG15^−/−^ neonates display elevated cytokine levels during CHIKV infection. Nine day old WT, IFNAR^−/−^, UbE1L^−/−^ and ISG15^−/−^ mice were injected with 2×10^5^ PFU CHIKV s.c. (**A**) The skin at the site of infection was harvested and the expression of IFNβ mRNA was evaluated by qRT-PCR. Vertical bars represent mean with standard error of the mean. (**B and C**) Serum was collected at the indicated time post-infection. (**B**) IFNα levels were quantified using an ELISA, vertical bars represent mean with standard error of the mean. Statistical comparison between the three genotypes via Mann-Whitney and Kruskal-Wallis test was not significant for both (A) and (B). **(C)** Cytokine and chemokine levels were measured by Bioplex assay (Mann-Whitney * p<0.05, ** p<0.005). Vertical bars represent standard error of the mean.

Since our evaluation of human infants and neonatal mice demonstrated that CHIKV infection induces a robust proinflammatory cytokine and chemokine response (**Figures 1** **,** **3**), we investigated the impact of ISG15 deficiency on this host response. IFNβ mRNA levels were induced in the skin of WT, UbE1L^−/−^ and ISG15^−/−^ mice at 24 and 48 hrs post infection with no significant differences noted between the three strains of animals (**Figure 8A**). Serum from WT, UbE1L^−/−^ and ISG15^−/−^ mice collected at 1 or 2 days post-infection were analyzed for IFNα as well as proinflammatory cytokines and chemokines. Analysis of IFNα serum levels also revealed no significant differences between WT, ISG15^−/−^, and UbE1L^−/−^ mice at either 24 or 48 hrs post-infection (**Figure 8B**). Despite similar viral loads and type I IFN induction, the ISG15^−/−^ mice displayed elevated levels of TNFα, IL-1β and IL-6 as compared to both the WT and UbE1L^−/−^ mice (**Figure 8C**). Interestingly the levels of these three pyrogenic cytokines in the ISG15^−/−^ mice were comparable to what was observed in the IFNAR^−/−^ pups, despite the latter having significantly higher viral loads. ISG15^−/−^ mice also displayed elevated chemokine levels, including CCL2, CCL3 and CCL5 (**Figure 8C**). Therefore, although viral titers between ISG15^−/−^, UbE1L^−/−^ and WT mice are similar, ISG15^−/−^ neonates display an exaggerated proinflammatory cytokine response to CHIKV infection. Together, these data indicate that ISG15, independent of UbE1L mediated conjugation, is contributing to the control of CHIKV infection by blunting potentially pathologic levels of innate effector molecules.

## Discussion

The ongoing epidemic of Chikungunya virus occurring in the Indian Ocean region has highlighted how little we understand about the pathogenesis of this virus. Epidemiological studies have provided the first documentation of vertical transmission, as well as providing detailed information about the severity of disease and long term sequelae [4], [46]–[48]. One important finding from these studies concerns the increased susceptibility of neonates and infants to severe forms of Chikungunya disease [10], [44], [49]. In this study, we evaluated the response of infants to CHIKV infection using data from both human samples collected during the La Réunion outbreak, as well as taking advantage of a newly described mouse model of infection. Our results show that human infants and murine neonates mount a robust innate immune response to CHIKV infection, which includes the induction of type I IFNs, several cytokines and chemokines, and the induction of at least a subset of IFN induced genes, including ISG15. We establish a role for ISG15 in the pathogenesis of CHIKV infection with an absolutely essential role in the neonatal response to infection. Moreover, the reported data suggest that ISG15 acts independent of UbE1L mediated conjugation, and rather than exerting a direct anti-viral role, it appears to be implicated in limiting an exaggerated inflammatory response.

In general, neonates are more susceptible to microbial and viral infection. This vulnerability has been explained by two principal mechanisms: broader tropism of the infectious agent or a defective host response. Regarding the latter, many argue that neonatal susceptibility to infection is due to a delayed or weaker immune response [16], [50]. Factors contributing to this include delays in immune system maturation, decreased expression of activation receptors, or distinct regulation of signaling pathways in neonatal vs. adult immune cells. Since the type I IFN response is critical for controlling CHIKV infection [15], [20], we hypothesized that neonates may have a defect in their ability to either produce and/or respond to IFN. Instead, we observed that the production of type I IFN was intact in both human infants and mouse neonates. Furthermore, the relative level of IFN produced in infants was higher than the responses observed in adult human patients, even when viral load was normalized between the two patient groups (**Figures 1** **–** **2**). These data indicate that neonates do not have a defect in their ability to produce type I IFN during CHIKV infection, and may actually be hyper-responsive. Very little has been reported on the signaling through RLRs or other viral sensors in neonates. Most previous work has supported the notion that signaling through TLRs, including TLR4, are diminished in neonates [51], [52]. However, one study did find that neonatal mice exhibited increased lethality following LPS treatment, due in part to an exaggerated pro-inflammatory cytokine response as compared to adult mice [53]. Future work is needed to characterize differences that may exist between neonatal and adult RLR signaling.

We also demonstrate that neonates can respond to the IFN that is produced. In both human infants and in neonatal mice, several known IFN-induced chemokines and cytokines were upregulated during the course of infection (**Figures 1** **–** [Fig ppat-1002322-g002] **3**). These results suggest that the ability to respond to IFNs was at least partially intact. These observations were confirmed by demonstrating the greater susceptibility of neonatal mice lacking expression of the type I IFN receptor (**Figure 4**); notably, the kinetics of viral replication and death were massively accelerated as compared to age-matched WT controls. Additionally, we demonstrate that the prophylactic exposure to an IFN-inducing adjuvant protected animals from challenge with lethal doses of CHIKV (**Figure 5**).

To provide insight into the mechanism by which IFN participates in the host response to CHIKV infection, we evaluated the role of ISG15, an anti-viral host protein that has previously been shown to be important in the control of several viruses, including SINV [34]. We found that ISG15^−/−^ mice were more susceptible to CHIKV infection, demonstrating a dramatic increase in lethality as compared to WT mice (**Figure 6A**). Moreover, ISG15 played a critical role in pIC induced protection of mice in a prophylactic setting (**Figure 6D, 6E**). While OAS has previously been shown to inhibit viral replication when over-expressed *in vitro* [54], to our knowledge our data is the first *in vivo* demonstration of an IFN effector molecule having activity against CHIKV.

Most importantly, the results from our current study indicate that ISG15 is regulating CHIKV pathogenesis by a unique mechanism of action. First, we demonstrate that ISG15 regulates CHIKV infection independent of UbE1L mediated conjugation. The protection mediated by ISG15 during pIC prophylaxis also appeared to be UbE1L independent, as UbE1L^−/−^ mice displayed survival curves similar to WT mice (**Figure 6D, 6E**). These results suggest that the non-classical function of ISG15 is at work both during acute viral infection and in the pIC induced protection seen in our mice. This is in contrast to both influenza virus and SINV infection, where the anti-viral activity of ISG15 is dependent upon ISG15 conjugation and abrogated in UbE1L^−/−^ animals [40], [41]. To date, UbE1L is the only known E1 for the ISG15 pathway. A second E1 has recently been identified for the ubiquitin pathway [55], leaving open the possibility that another E1 may exist for ISG15. However, our current analysis (**Figure 6**) and previous studies [39] have revealed no conjugation activity in UbE1L^−/−^ cells and mice. Therefore the actions of ISG15 during CHIKV infection are likely to be independent of conjugation, and mediated by free ISG15. Second, it appears that during CHIKV infection, ISG15 is not functioning as a direct antiviral molecule. In both the influenza and SINV models, the increase in lethality was accompanied by a dramatic increase in viral loads [34], [38], [41]. In contrast, during CHIKV infection, ISG15^−/−^ mice did not show an increased CHIKV burden (**Figure 7**). Instead, we noted a significant elevation of several proinflammatory cytokines and chemokines in the ISG15 deficient mice (**Figure 8**). Therefore, as opposed to having direct antiviral activity, it appears that ISG15 modulates the immune response during CHIKV infection. Finally, in contrast to what was previously reported for control of SINV infection [34], a recombinant CHIKV expressing ISG15 was unable to rescue neonatal ISG15^−/−^ mice from viral induced lethality (**Figure S4**). The inability to rescue the ISG15^−/−^ phenotype may be due to insufficient levels or inappropriate timing of ISG15 expression; or alternatively, may indicate that ISG15 expression is required in an uninfected cell. Since we detect no differences in viral load, and instead observed increased cytokine levels in the ISG15^−/−^ mice, we favor this latter possibility. Further analysis into the precise mechanism by which ISG15 regulates the host response to CHIKV should provide additional insight into this issue. Together, our data suggest a novel mechanism for ISG15, which is likely to be independent of conjugation and extrinsic to virally infected cells.

The precise mechanism by which ISG15, independent of UbE1L mediated conjugation, contributes to the control of viral infection is currently unclear. The most intriguing difference we have noted to date is the increased cytokine responses in the mice lacking ISG15 (**Figure 8**). As noted above, while we cannot formally exclude the possibility of another E1 functioning in this system, it seems most likely that free, unconjugated ISG15 mediates the activity during CHIKV infection. Free ISG15 is found within the cell, but interestingly it may also be secreted by a still undefined mechanism [33]. Previous work has shown that unanchored ISG15 can associate non-covalently with proteins (i.e. independent of conjugation). For example, the NS1 protein of influenza B virus can non-covalently bind ISG15, thereby inhibiting its interaction with UbE1L and blocking conjugation of target proteins [24]. The over-expression of ISG15 has also been shown to disrupt Nedd4 ligase activity and inhibit Ebola virus VLP release [56], [57]. Recent research within the ubiquitin field has described a role for unanchored polyubiquitin chains, shown to regulate TRAF6 function, as well as promote RIG-I dimerization and signaling [58], [59]. It is therefore possible that intracellular, unanchored ISG15 interacts non-covalently with members of an innate immune signaling pathway to regulate cytokine and chemokine production or other host response pathways. Alternatively, released ISG15 could be contributing to the phenotype seen during CHIKV infection. Indeed, the 17 kDa form of ISG15 is released into the serum in both WT and UbE1L^−/−^ mice during CHIKV infection (**Figure 6**). Released ISG15 has been reported to function as an immunomodulatory molecule, increasing NK cell proliferation and lytic activity, acting as a neutrophil chemoattractant, and upregulating E-cadherin expression on dendritic cells [35]–[37]. Released ISG15 could function as in immunomodulatory cytokine by signaling through a receptor to regulate the cytokine response or through its ability to function as a chemoattractant. In order to characterize these effects in greater detail, a receptor for ISG15 must be identified. Future studies evaluating these possibilities will be required in order to further define the mechanism by which ISG15 is contributing to the host response to CHIKV.

In conclusion, we have demonstrated that neonates are capable of producing type I IFN in response to CHIKV, which serves to limit viral infection though remains insufficient to clear the virus. We have demonstrated a critical, age-dependant role for ISG15 during neonatal infection. We have also characterized the mechanism of ISG15 activity, revealing a novel mechanism for ISG15, independent of UbE1L mediated conjugation, and functioning as a putative immunomodulator of proinflammatory cytokines. The ability of pIC to protect neonatal mice against CHIKV infection suggests that manipulation of the IFN signaling pathway, and perhaps the induction of ISG15, may be an appropriate therapeutic target for combating CHIKV infection.

## Materials and Methods

### Ethics statement

All human studies were approved by the Committee for Clinical Research at the Institut Pasteur, project number RBM 2009-23, on July 9, 2009. Written informed consent was obtained from the study participants or legal guardians. For mouse studies at the Institut Pasteur and at Washington University, the principles of good laboratory animal care were carried out in strict accordance with the recommendations in the Guide for the Care and Use of Laboratory Animals of the National Institutes of Health and following the International Guiding Principles for Biomedical Research Involving Animals. The protocols were approved by the Animal Studies Committee at Washington University (#20090287) and the Institutional Committees on Animal Welfare of the Institut Pasteur (OLAW assurance # A5476-01). All efforts were made to minimize suffering.

### Human study

Retrospective study on patients who presented to the emergency or pediatric service of the Groupe Hospitalier Sud Réunion, Saint Pierre, La Réunion, France from January 2006 through May 2006. Sera samples were collected and stored at −80°C. CHIKV infection was confirmed by RT-PCR. All patients were negative for anti-CHIKV IgG and IgM as assessed by immunocapture Elisa. The control cohort consisted of patients that presented to the orthopedic chirurgy or infant chirurgy service of the Groupe Hospitalier Saint Pierre before the epidemic. These patients were IgG, IgM and RT-PCR negative for CHIKV. Data from adult patients were previously reported [20], but are shown here for comparison to data from infected neonates.

### Virus

The preparation of CHIKV from clinical samples has been previously described [60]. CHIKV (06.21) strain was isolated during the epidemic in La Réunion and then propagated three time on C6/36 mosquito cells to generate a stock (6×10^7^ PFU/mL) that was used in all experiments.

#### Recombinant CHIKV strains

The generation and characterization of a recombinant double subgenomic CHIKV virus expressing GFP, CHIKV-LR 5′ GFP, has been previously described [61]. Recombinant CHIKV expressing ISG15 were generated as follows.

- ISG15 LRLRGG. Nucleotides 1 to 465 of murine ISG15 were PCR amplified using a 5′ primer that introduced an AscI restriction site and a 3′ primer containing GGT GGG TAA sequence and a PmeI site.
- ISG15 LRLRAA. Nucleotides 1 to 465 of murine ISG15 were PCR amplified using a 5′ primer with an AscI restriction site and a 3′ primer containing GCG GCG TAA sequence and a PmeI site.

The correct sequence for each virus was confirmed. Generation of recombinant ISG15 viruses was previously described [62]. Viral stocks were generated by in vitro transcription of linearized cDNA templates, followed by transfection of the transcripts with Lipofectamine (Invitrogen) into BHK cells. Supernatents were harvested 48 hrs post transfection and viral titers were determined by plaque assay as described below. To examine growth characteristics of the recombinant viruses, BHK cells were infected at a multiplicity of infection (MOI) of 1.0 and viral titers were measured at various times post-infection. To determine the expression of ISG15 from recombinant viruses, BHK cells were infected at an MOI = 1.0 and at various times post-infection total cell lysates were harvested and ISG15 expression was analyzed by Western blot analysis.

### Mice

Experiments in mice were carried out at both the Institut Pasteur, Paris, France and at Washington University School of Medicine, St. Louis, Missouri, using the identical CHIKV viral stock described above. Infection of 9 day old pups with 2×10^5^ PFU subcutaneously (s.c.) resulted in approximately 50–60% lethality in WT mice at both locations. For experiments carried out at the Institut Pasteur, eight day old C57BL/6 litters were obtained from Charles River laboratories (France). For experiments carried out at Washington University, mice were maintained at Washington University School of Medicine in accordance with all federal and University guidelines. ISG15^−/−^ mice were provided by Dr. Ivan Horak and Dr. Klaus-Peter Knobeloch. UbE1L^−/−^ mice were provided by Dr. Dong-Er Zhang. The generation of both the ISG15^−/−^ and UbE1L^−/−^ mice has been previously described [39], [45]. C57BL/6, IFNαβ receptor 1 (IFNAR^−/−^), UbE1L^−/−^ and ISG15^−/−^ mice, all on the C57BL/6 background, were bred and maintained in our mouse colony. Congenic SNP analysis (Taconic laboratories) of UbE1L^−/−^ and ISG15^−/−^ mice confirmed that these mice were fully backcrossed, with 99.93% and 99.72% identity to C57BL/6 mice, respectively. For neonatal experiments mice were infected between 9 and 12 days of age as indicated. Litters were weight matched at the initiation of the experiments. For adult experiments, mice were infected between 6–8 weeks of age and were age and sex matched within experiments.

### Viral studies

For neonatal experiments using the clinical isolate of CHIKV, 9 day old pups were infected with 2.0×10^5^ PFU CHIKV in 20–30 µL of PBS s.c. into the right flank. Infected mice were followed daily for weight gain, signs of disease, and lethality for 21 days post-infection. Paralysis was scored as an inability or long delay (>5s) to return and land on its feet when flipped on its back. For experiments with adult mice, 6–8 week old mice were infected as outlined above and followed for daily weight loss and lethality. For experiments utilizing the recombinant CHIKV clones, 6 day old pups were infected with 3×10^5^ PFU of the indicated recombinant virus diluted in 30 µL of PBS by s.c. injection into the right flank. Viral titers were determined in organs harvested at the indicated days post-infection. Organs were harvested into 1 ml of DMEM without fetal bovine serum and homogenized with 1.0 mm diameter zirconia-silica beans at 3,200 rpm for 1 minute with a MagnaLyzer prior to plaque assay on BHK cells, protocol modified from [63]. Serial dilutions of organ homogenates in DMEM with 1% FBS was added to BHK cells (6×10^5^ cells for 6 well plates) and incubated for 1 hr at 37°C. An agar overlay was then added to the cells and incubated for 28 hrs at 37°C. Plates were fixed with 1% formaldehyde (>30 min at room temperature), agar plugs were removed and plaques were visualized using a 1% crystal violet solution.

### Poly I∶C prophylactic treatment studies

Eight day old mice were injected intraperitoneally (i.p.) with 10–25 µg of high molecular weight pIC (Invivogen). Twenty-four hours later, mice were challenged with 2×10^5^ PFU CHIKV s.c. Mice were monitored daily for the development of symptoms and followed for lethality as described above.

### Cytokine analysis

Sera were harvested and conserved at −80°C for analysis. Human cytokines were measured by Luminex (25 plex kits, Biosource, Invitrogen) following manufacturer's instructions. Human CXCL10 was re-titrated by ELISA (human quantikine ELISA kit, R&D). Mouse sera were obtained after coagulation of blood in T-MG tubes (Terumo). Mouse IFNα levels were quantified by ELISA (PBL biomedical) and other cytokines were measured using Luminex technology with either the 32 plex from Millipore (MPXMCYTO-70X) or by customized 10 plex from Biorad.

### qRT-PCR for quantification of viral load and IFN mRNA

For determination of patient viral load, total nucleic acid extraction was performed on sera in a MagNa Pure automate using the Total Nucleic Acid Kit (Roche Diagnostics). CHIKV RNA was detected with specific taqman probes using a one step RT-PCR (Master RNA hybridization probes, Roche) performed on a Chromo 4 machine (Biorad). The 20 µL reaction mix contained 2 µL of extracted RNA, 7.5 µL of LightCycler RNA Master Hyb-Probe, 3.25 mmol/L Mn_2_, 450 nmol/L CHIKV-forward primer, 150 nmol/L CHIKV-reverse primer, 150 nmol/L CHIKV Probe (5 6-carboxyfluorescein-3 TAMRA) (TibMol-Biol). The thermal cycling consisted of a reverse transcription at 61°C for 20 min followed by 45 cycles at 95°C for 5 s and 60°C for 15 s. The fluorescence was measured at 530 nm. CHIKV load is measured using a synthetic RNA calibrator [64]. CHIKV-rev CCAAATTGTCCGGGTCCTCCT; CHIKV-forw AAGCTCCGCGTCCTTTACCAAG; Probe: Fam-CCATGTCTTCAGCCTGGACACCTTT-TAMRA.

For mouse studies, skin tissue was harvested at the site of infection on days 1 and 2 post-infection. Tissue was snap frozen in liquid nitrogen and then homogenized in RLT+ with 0.04 M DTT, and RNA was extracted with the Qiasymphony robot (Qiagen) with a protease step and a DNase step. The quality and quantity of RNA was evaluated with the Agilent technology, with the RNA integrity number between 8 and 9.5. Reverse transcription was performed with random primers (Roche) using Superscript enzyme (Invivogen). cDNA for murine IFN-β and ISG15 were detected using Applied Biosystem Taqman probes (Mm00439546-s1, Mm01705338-s1). To analyze the relative fold induction of mRNA, GAPD expression levels were determined in parallel for normalization using the CT method.

### Western blot analysis

Nine day old mice were infected with 2×10^5^ PFU CHIKV. Tissue homogenates as well as serum samples were subjected to protein electrophoresis on a 12% Tris gel. The gel was transferred to a polyvinylidene fluoride membrane and probed for ISG15 expression using a rabbit anti-ISG15 polyclonal serum (1∶5000) as previously described [62]. The membrane was then developed with a horseradish peroxidase (HRP)-conjugated goat anti-rabbit antiserum (Jackson Immunoresearch, West Grove, Pennsylvania) diluted 1∶200,000. For loading controls, the same blot was re-probed with anti-β-actin mAb (clone AC-74; Sigma) and then developed with a HRP conjugated goat anti-mouse antibody (Jackson Immuno-Research). All blots were developed with chemiluminescent reagent (Millipore).

### Flow cytometry

Spleens were harvested from naïve nine day old WT, UbE1L^−/−^ and ISG15^−/−^ mice (12 mice per strain). Lymphocyte subsets were stained using the following cell surface markers: CD3, CD4, CD8, NK1.1, CD19, F4/80 and Gr1. Data is represented as percent of the total cell population and Kruskal-Wallis test was used to compare the three genotypes.

### Statistical analysis

Human data was analyzed using the OMNIVIZ statistical platform (BioWisdom, Cambridge, UK) to perform comparisons among data sets using nonparametric tests (Mann-Whitney U-test) and false discovery rate (FDR) procedures, a permutation-based method to correct for the increased probability of obtaining a false positives among all significant tests [65]. Additional data was analyzed using the Prism software (Graphpad software). Differences were considered significant if p<0.05.

### List of accession numbers in GenBank

IFNγ: NM_008337, CCL2: NM_011333, CCL4: NM_013652, CXCL9: NM_008599, CXCL10: NM_021274, IL12: NM_008352, IL1Rα: NM_001039701, IL4: NM_021283, IL5: NM_010558, IL13: NM_008355, IL15: NM_008357, IL17: NM_010552, IFNβ: NM_010510, IRF7: NM_016850, Mx1: NM_010846, ISG15: NM_015783, IL1β: NM_008361, IL6: NM_031168, TNFα: NM_013693, IFNAR1: NM_010508, UbE1L: NM_023738.

## Supporting Information

Figure S1 **Pre-treatment with IFN**β **improves the outcome of CHIKV infection.** Mice 8 days of age were injected i.p. with 5000 U of IFNβ and 24 hours later mice were infected s.c. in the right flank with 2×10^5^ PFU CHIKV. Mice were scored for clinical signs of disease on days 7, 11 and 18 post-infection. The ability of the mice to return and land on its feet when flipped over was assessed. Paralysis was defined as the inability or delayed time to return (>5 s). 3 independent experiments are compiled.
(PPT)

Figure S2 **Induction of ISG15 during neonatal CHIKV infection is largely dependent upon IFNAR1 signaling.** Serum from nine day old WT, UbE1L^−/−^, ISG15^−/−^ and IFNAR^−/−^ mice infected s.c. with 2×10^5^ PFU CHIKV was collected on day 1 post-infection and ISG15 expression was assessed by western blot.
(PPT)

Figure S3 **The role of ISG15 during CHIKV infection is age dependent.** WT, UbE1L^−/−^ and ISG15^−/−^ mice were infected with 2×10^5^µ PFU CHIKV s.c. at either **(A)** eleven days of age, **(B)** twelve days of age, or **(C)** 6–8 weeks of age and were monitored for survival for 21 days post-infection. Kaplan-Meier survival curves are shown.
(PPT)

Figure S4 **Recombinant CHIKV viruses expressing WT ISG15 do not rescue ISG15** ^−**/**−^ **mice.** Recombinant CHIK viruses were generated to express the following proteins: wild type ISG15(−LRLRGG), non-conjugatable ISG15(−LRLRAA), and GFP(−GFP). **(A)** Schematic representation of recombinant CHIK clones adapted from [61]. **(B and C)** BHK cells were infected with the indicated rCHIK viruses at an MOI = 1. (B) Viral titers were measured at 0,6,12,24,48 hrs post-infection by plaque assay. (C) Cell lysates were collected at 0,6,12, and 24 hrs post-infection and were analyzed for ISG15 expression by western blot. **(D)** Six day old ISG15^−/−^ mice were infected with either CHIK-GFP or CHIK-LRLRGG at 3×10^5^ PFU s.c. Mice were monitored for lethality for 21 days with data displayed as Kaplan-Meier curves.
(PPT)

Table S1 **Clinical signs in adult and infant cohort.**
(PPT)

Table S2 **Splenic lymphocyte subsets in naïve WT, UbE1L** ^−**/**−^ **and** **ISG15** ^−**/**−^ **neonatal mice (% total cell population, mean ± sem).**
(PPT)
