# Supplementary figures and images for: ISG15 Is Critical in the Control of Chikungunya Virus Infection Independent of UbE1L Mediated Conjugation
Source: PLoS Pathog. 2011 Oct 20;7(10):e1002322. doi: 10.1371/journal.ppat.1002322 (PMC3197620; doi:10.1371/journal.ppat.1002322)

## Slide 1
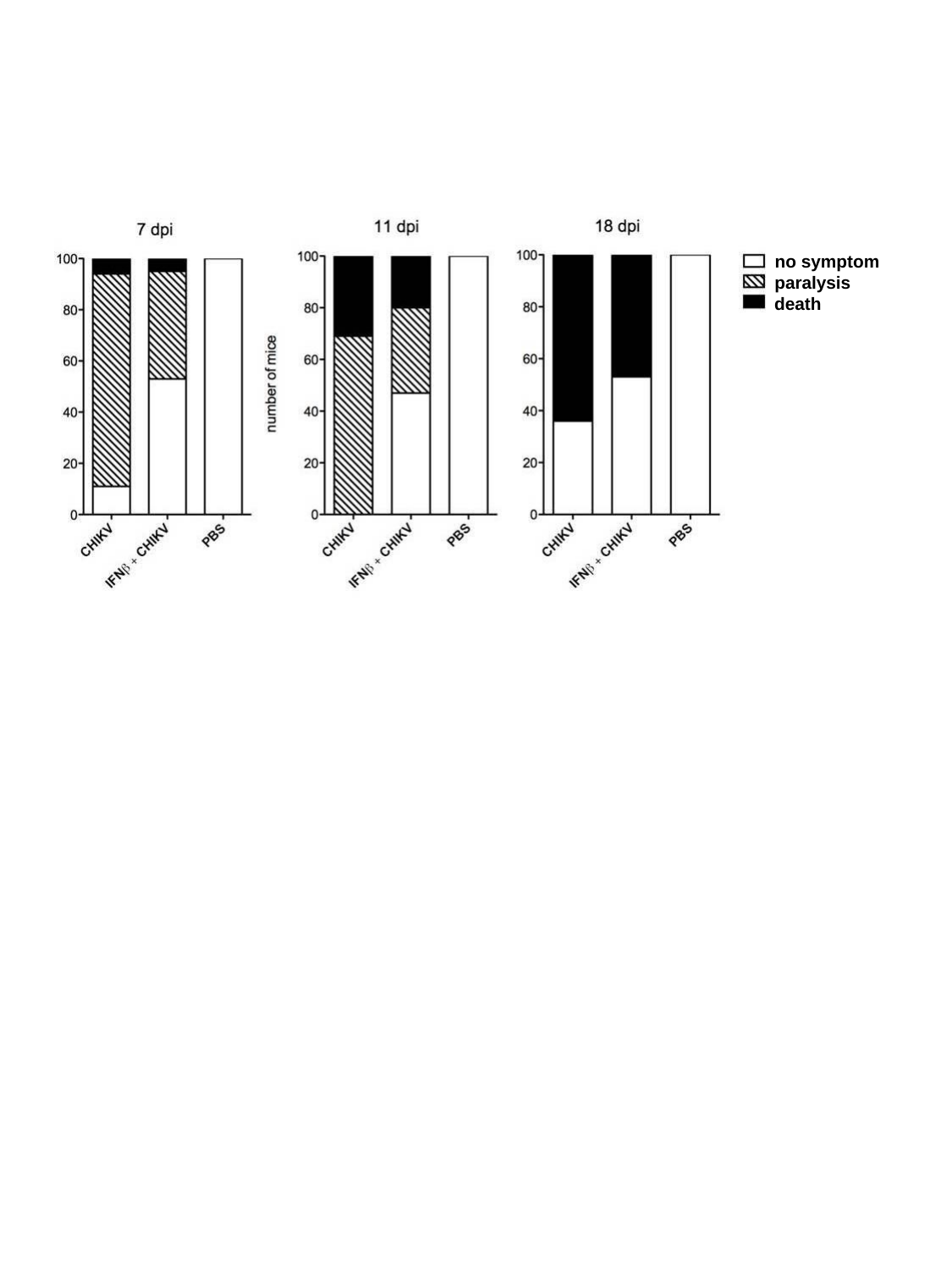

no symptom
paralysis
death

Supplement: Figure S1 — Pre-treatment with IFNβ improves the outcome of CHIKV infection. Mice 8 days of age were injected i.p. with 5000 U of IFNβ and 24 hours later mice were infected s.c. in the right flank with 2×10^5^ PFU CHIKV. Mice were scored for clinical signs of disease on days 7, 11 and 18 post-infection. The ability of the mice to return and land on its feet when flipped over was assessed. Paralysis was defined as the inability or delayed time to return (>5 s). 3 independent experiments are compiled. (PPT) [file ppat.1002322.s001.ppt]

## Slide 1
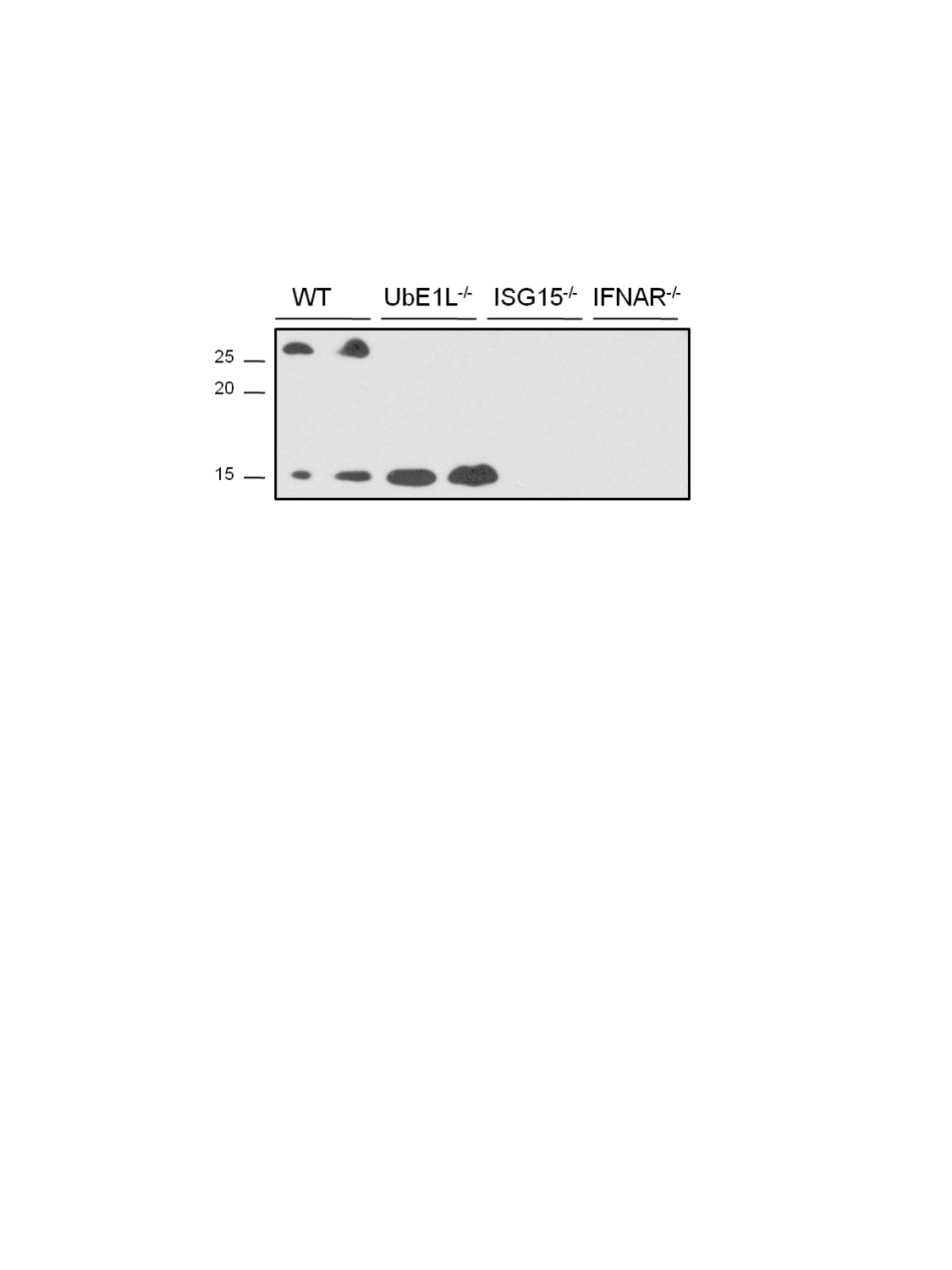

Supplement: Figure S2 — Induction of ISG15 during neonatal CHIKV infection is largely dependent upon IFNAR1 signaling. Serum from nine day old WT, UbE1L−/−, ISG15−/− and IFNAR−/− mice infected s.c. with 2×10^5^ PFU CHIKV was collected on day 1 post-infection and ISG15 expression was assessed by western blot. (PPT) [file ppat.1002322.s002.ppt]

## Slide 1
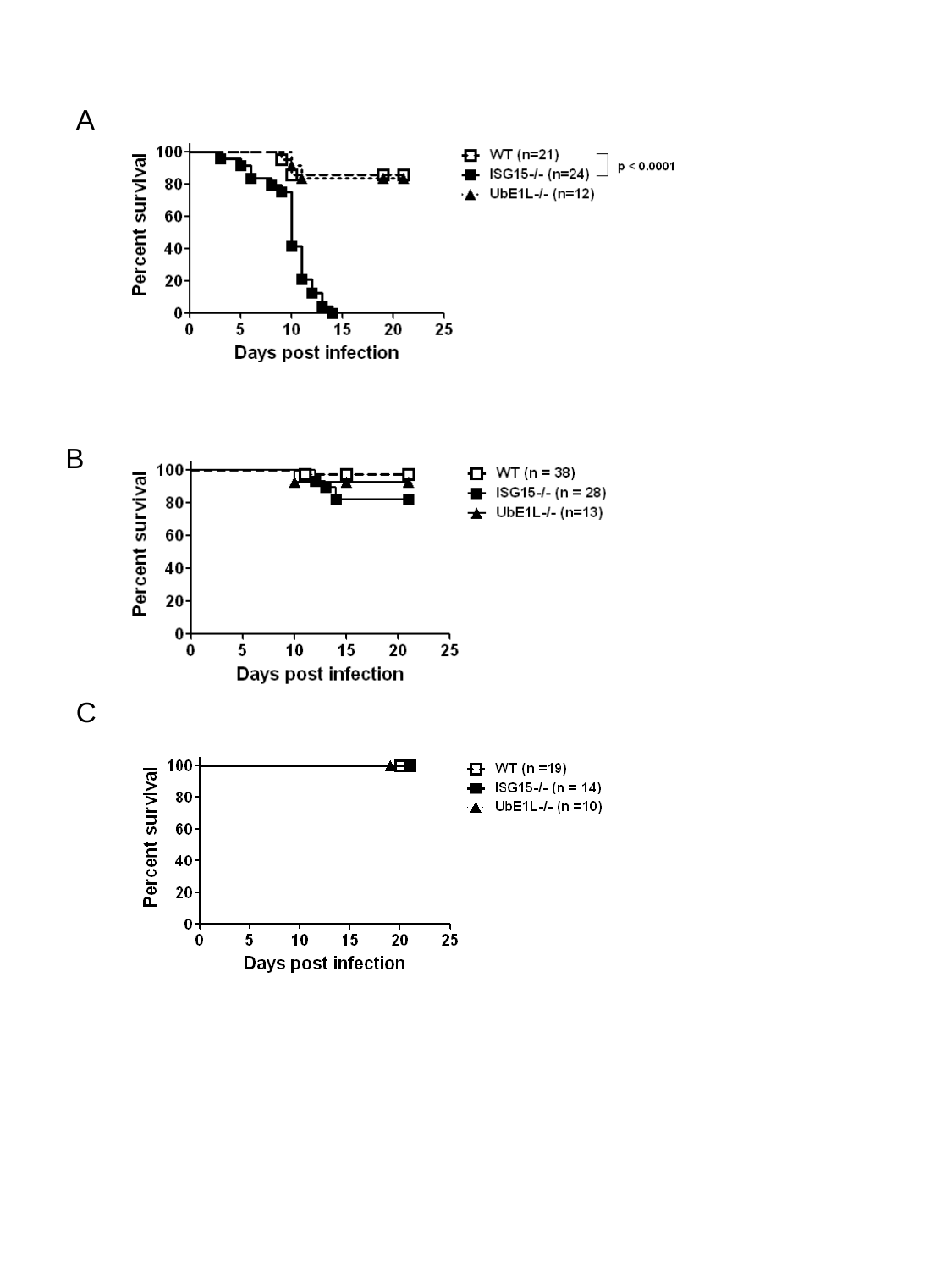

A
B
C

Supplement: Figure S3 — The role of ISG15 during CHIKV infection is age dependent. WT, UbE1L−/− and ISG15−/− mice were infected with 2×10^5^µ PFU CHIKV s.c. at either (A) eleven days of age, (B) twelve days of age, or (C) 6–8 weeks of age and were monitored for survival for 21 days post-infection. Kaplan-Meier survival curves are shown. (PPT) [file ppat.1002322.s003.ppt]

## Slide 1
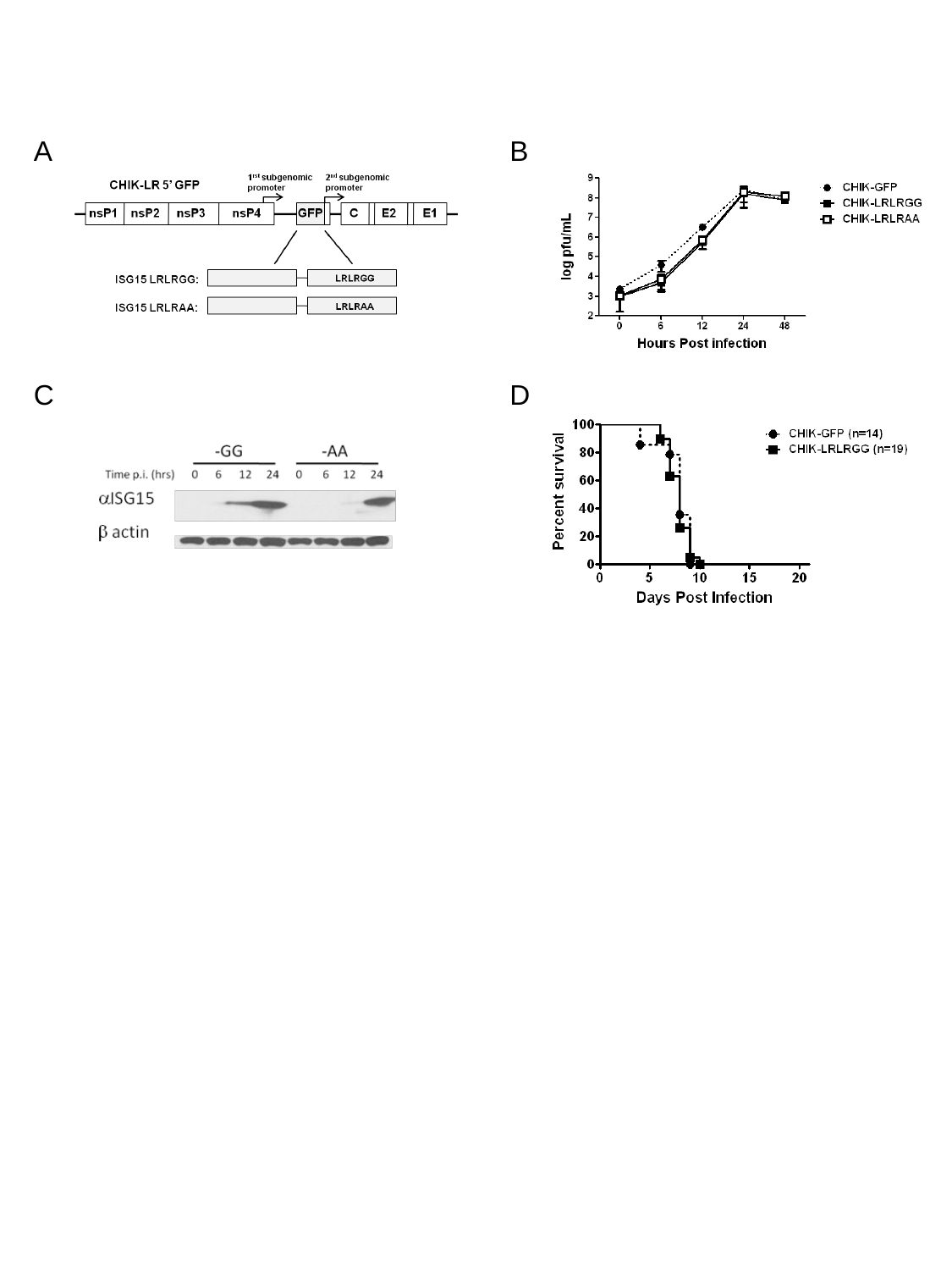

A
B
C
D

Supplement: Figure S4 — Recombinant CHIKV viruses expressing WT ISG15 do not rescue ISG15 −/− mice. Recombinant CHIK viruses were generated to express the following proteins: wild type ISG15(−LRLRGG), non-conjugatable ISG15(−LRLRAA), and GFP(−GFP). (A) Schematic representation of recombinant CHIK clones adapted from [61]. (B and C) BHK cells were infected with the indicated rCHIK viruses at an MOI = 1. (B) Viral titers were measured at 0,6,12,24,48 hrs post-infection by plaque assay. (C) Cell lysates were collected at 0,6,12, and 24 hrs post-infection and were analyzed for ISG15 expression by western blot. (D) Six day old ISG15−/− mice were infected with either CHIK-GFP or CHIK-LRLRGG at 3×10^5^ PFU s.c. Mice were monitored for lethality for 21 days with data displayed as Kaplan-Meier curves. (PPT) [file ppat.1002322.s004.ppt]

## Slide 1
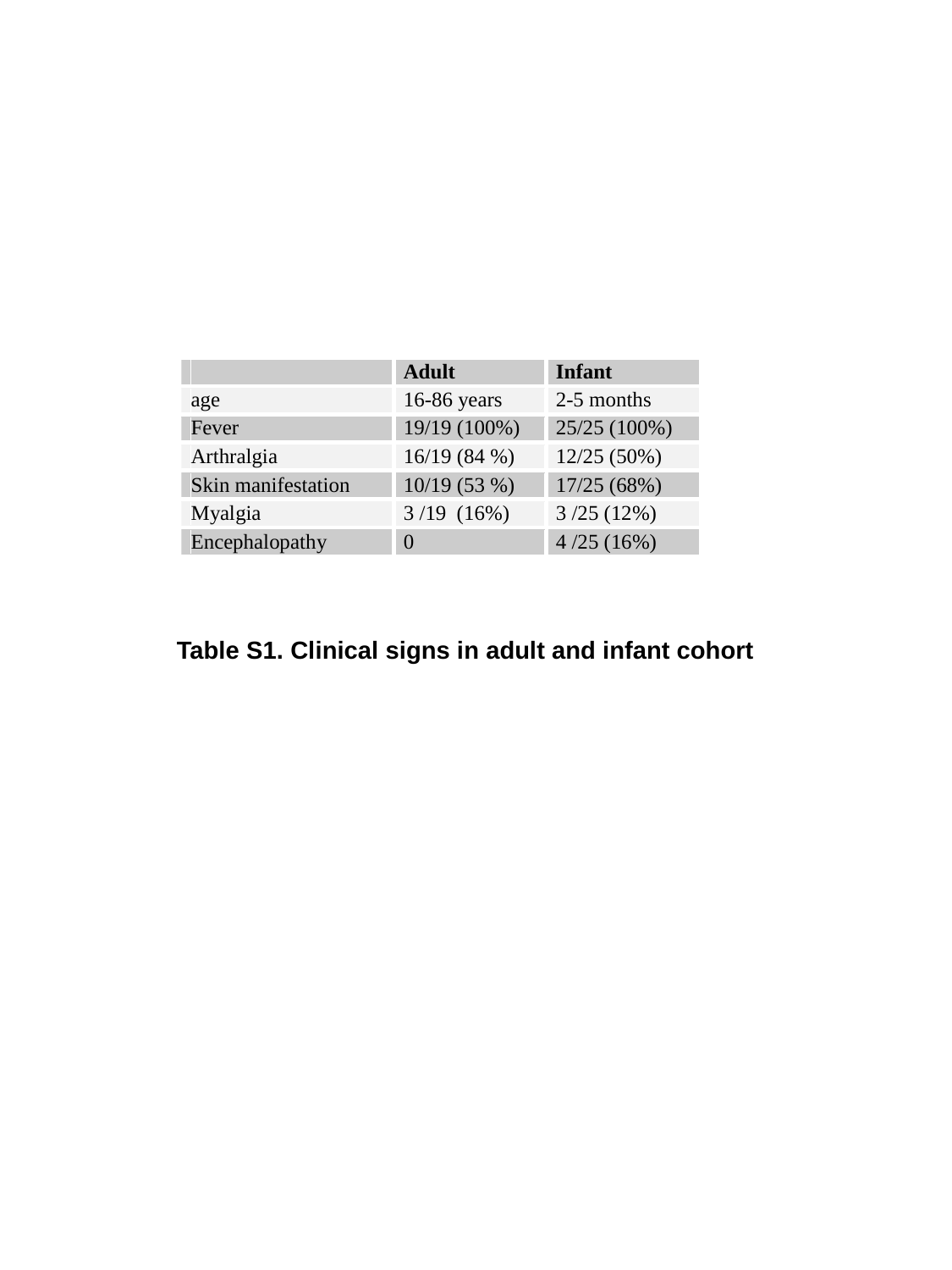

Table S1. Clinical signs in adult and infant cohort

Supplement: Table S1 — Clinical signs in adult and infant cohort. (PPT) [file ppat.1002322.s005.ppt]
