# Supplementary material for: ISG15 Is Critical in the Control of Chikungunya Virus Infection Independent of UbE1L Mediated Conjugation
Source: PLoS Pathog. 2011 Oct 20;7(10):e1002322. doi: 10.1371/journal.ppat.1002322 (PMC3197620; doi:10.1371/journal.ppat.1002322)
Supplement: Table S2 — Splenic lymphocyte subsets in naïve WT, UbE1L −/− and ISG15 −/− neonatal mice (% total cell population, mean ± sem). (PPT) [file ppat.1002322.s006.ppt]

## Slide 1
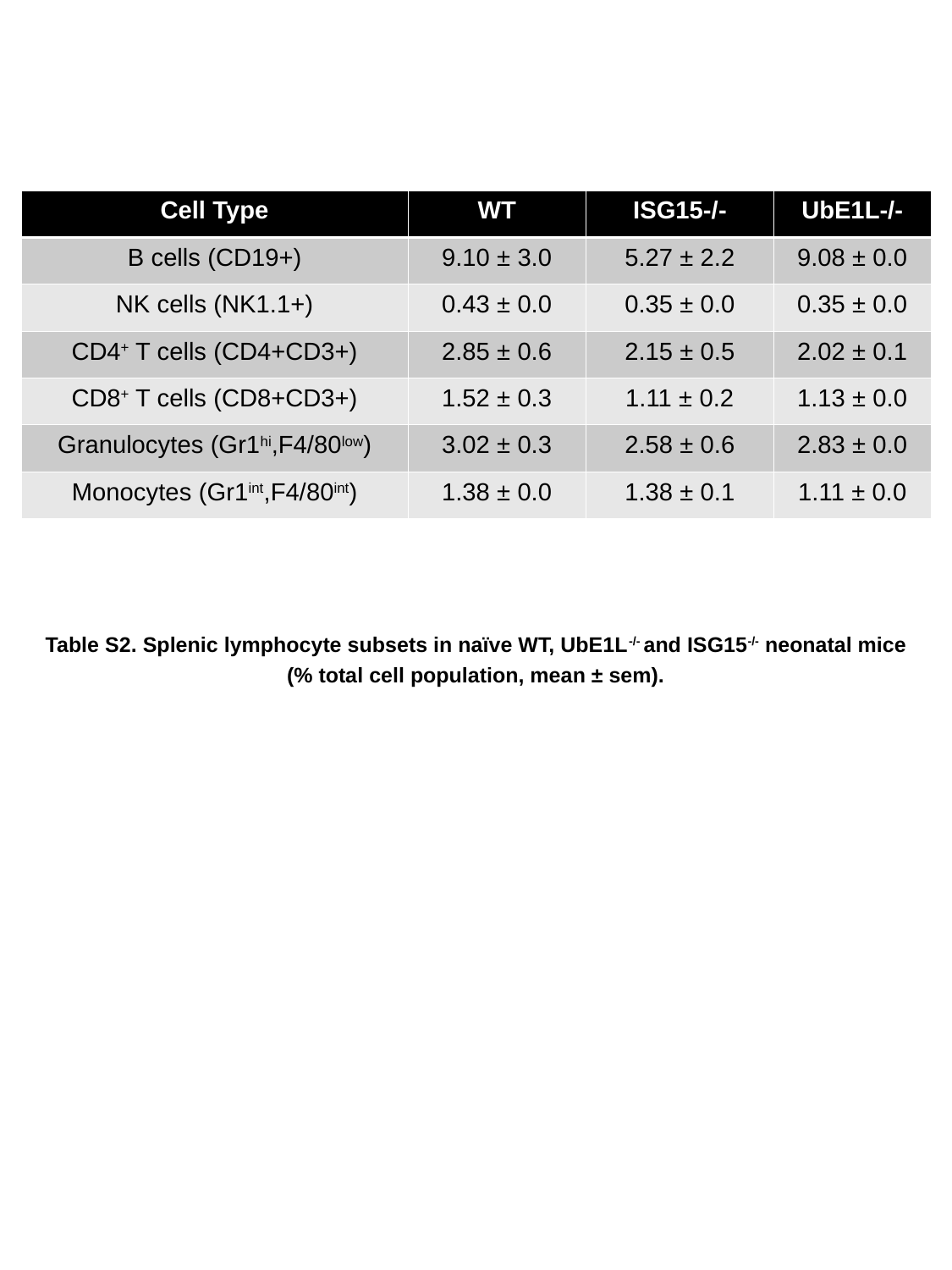

| Cell Type | WT | ISG15-/- | UbE1L-/- |
| --- | --- | --- | --- |
| B cells (CD19+) | 9.10 ± 3.0 | 5.27 ± 2.2 | 9.08 ± 0.0 |
| NK cells (NK1.1+) | 0.43 ± 0.0 | 0.35 ± 0.0 | 0.35 ± 0.0 |
| CD4+ T cells (CD4+CD3+) | 2.85 ± 0.6 | 2.15 ± 0.5 | 2.02 ± 0.1 |
| CD8+ T cells (CD8+CD3+) | 1.52 ± 0.3 | 1.11 ± 0.2 | 1.13 ± 0.0 |
| Granulocytes (Gr1hi,F4/80low) | 3.02 ± 0.3 | 2.58 ± 0.6 | 2.83 ± 0.0 |
| Monocytes (Gr1int,F4/80int) | 1.38 ± 0.0 | 1.38 ± 0.1 | 1.11 ± 0.0 |
Table S2. Splenic lymphocyte subsets in naïve WT, UbE1L-/- and ISG15-/- neonatal mice
 (% total cell population, mean ± sem).
